# Surgical Tricuspid Valve Repair Resulting in Acquired Gerbode Defect: A Rare and Challenging Case

**DOI:** 10.1016/j.jaccas.2026.107486

**Published:** 2026-03-19

**Authors:** Joseph Jebain, Mohamad Bader Abo Hajar, Mohammed Salih, Ahmed Elkaialy, Ghadi Moubarak, Eduardo Hernandez, Molly Szerlip, Karim Al-Azizi, Ralph Matar, Srinivasa Potluri

**Affiliations:** a Baylor Scott and White Research Institute, Dallas, Texas, USA; b Baylor Scott & White–The Heart Hospital, Plano, Texas, USA

**Keywords:** right-sided catheterization, tricuspid valve, valve repair, ventricular septal defect

## Abstract

**Background:**

Acquired Gerbode defect is a complication of cardiac surgery. Diagnostic challenges arise because of frequent mischaracterization as severe tricuspid regurgitation on imaging, often delaying definitive management.

**Case Summary:**

After undergoing surgical tricuspid valve repair, a patient developed an acquired Gerbode defect located in the membranous septum beneath the noncoronary cusp of the aortic valve. The defect remained undetected for over 3 years owing to misinterpretation of echocardiographic findings as severe tricuspid regurgitation. Comprehensive transesophageal echocardiography enabled accurate diagnosis and anatomical localization.

**Discussion:**

The defect was successfully closed percutaneously under transesophageal echocardiographic and fluoroscopic guidance using a 6-mm Amplatzer atrial septal defect occluder device. The procedure resulted in complete closure of the shunt, with no residual defect or complications.

**Take-Home Message:**

This case highlights the importance of advanced imaging for the diagnosis of elusive acquired Gerbode defects and demonstrates that percutaneous device closure is a safe and effective therapeutic option for this uncommon postsurgical complication.

## Graphical Abstract

**Figure:**
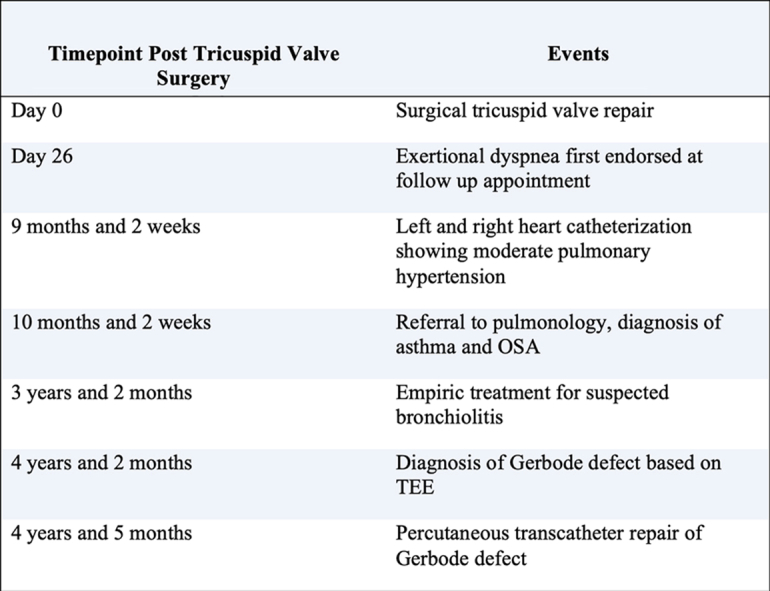

## Background

Acquired Gerbode defect, defined as an abnormal communication between the left ventricular outflow tract (LVOT) and the right atrium, is an uncommon form of intracardiac shunt that may arise as a complication of invasive cardiac procedures.^1^^,^^2^ Published cases of acquired Gerbode defects describe surgical aortic or mitral valve replacements, ventricular septal defect (VSD) closures, or endocarditis, but uncommonly tricuspid valve repair, as the underlying etiology.^1^, ^2^, ^3^, ^4^, ^5^, ^6^ Acquired Gerbode defect after tricuspid valve repair is particularly uncommon, with only isolated case reports and no established incidence rate.^1^^,^^2^ We herein describe an elusive case of acquired Gerbode defect after surgical tricuspid valve repair, highlighting our unique transcatheter approach to this diagnostic and therapeutic challenge.

**Take-Home Messages**

• Acquired Gerbode defects pose diagnostic challenges, making comprehensive TEE with 3D and Doppler imaging essential for accurate characterization and procedural planning.
• Percutaneous closure using a septal occluder is a safe, effective option in carefully selected patients.
• This case emphasizes the importance of early detection and continued case reporting to guide best practices.

## Case Presentation

A 79-year-old woman with a past medical history of atrial fibrillation with subsequent ablation including atrioventricular nodal ablation, complete heart block with subsequent biventricular pacemaker placement, and surgical tricuspid valve repair (#28 Flexible C annuloplasty band) for severe tricuspid regurgitation, experienced progressive dyspnea on exertion after her surgical tricuspid valve repair, despite completing cardiac rehabilitation. She first endorsed dyspnea with minimal exertion during an office visit only 26 days after the tricuspid valve repair.

The patient's dyspnea progressed to the point where she required home oxygen, with limitation in her activities of daily living. Multiple transthoracic echocardiography (TTE) as performed by her primary cardiologist consistently demonstrated moderate to severe tricuspid regurgitation with an eccentrically directed jet and severely elevated right ventricular systolic pressure (Video 1, Video 2). Left and right heart catheterization was then performed (9 months and 2 weeks after tricuspid valve surgery), demonstrating moderate pulmonary hypertension (right ventricular systolic pressure: 56 mm Hg). The patient was then referred to a pulmonologist and subsequently underwent pulmonary function testing and a sleep study.

Pulmonary function testing showed a mild obstructive pattern with air trapping consistent with asthma. The sleep study showed mild to moderate obstructive sleep apnea but with severe nocturnal hypoxemia. These were medically managed with bronchodilator/inhaled corticosteroid therapy and nightly continuous positive airway pressure. The patient's dyspnea was thought to initially improve, but ultimately worsened. She underwent computed tomography of the chest (3 years and 2 months after tricuspid valve surgery), which showed ground-glass opacities, and she was empirically treated for suspected bronchiolitis secondary to occult connective tissue disease with prednisone and azithromycin. However, her dyspnea persisted, and she began to show signs of volume overload, which was managed with furosemide.

**Figure 1:**
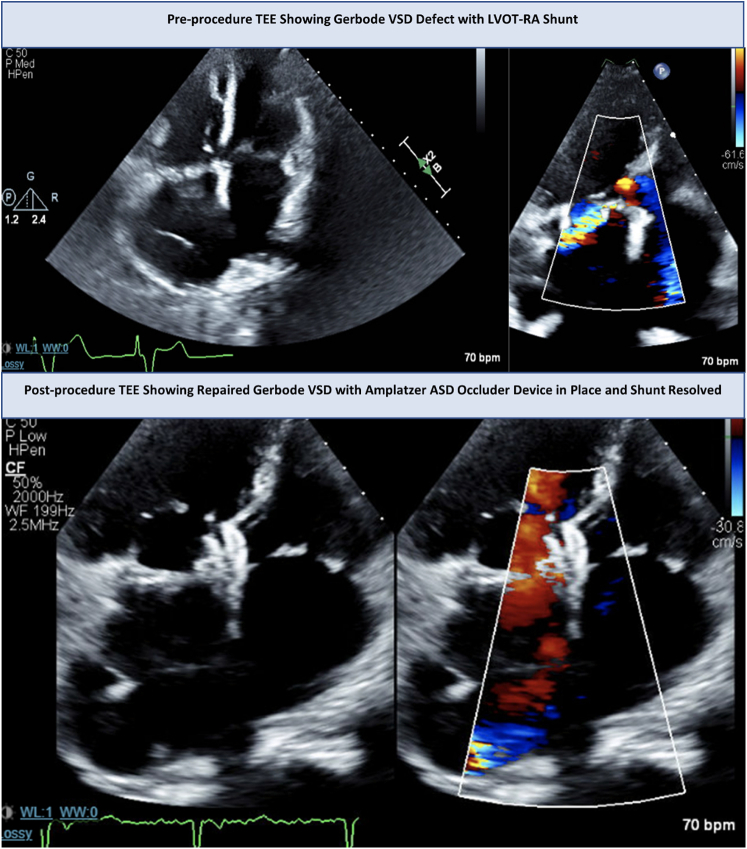
Preprocedure and Postprocedure Transesophageal Echocardiography ASD = atrial septal defect; LVOT-RA = left ventricular outflow tract–to–right atrium. TEE = transesophageal echocardiography

The patient was then referred to our pulmonary hypertension cardiology clinic. A repeat TTE finally captured a possible Gerbode defect, confirmed on follow-up transesophageal echocardiography (TEE), at 4 years and 2 months after the tricuspid valve surgery. This TEE demonstrated the presence of a 5-mm connection between the LVOT and the right atrium just underneath the noncoronary cusp of the aortic valve, consistent with a Gerbode defect with significant left-to-right shunt (the actual culprit of the pulmonary ground-glass opacities), rather than true tricuspid regurgitation (Figure 1). The defect was concluded to likely be an acquired defect from the prior tricuspid valve repair.

After discussion among members of a multidisciplinary heart team, a transcatheter approach to repair the defect was recommended. Four years and 5 months after the surgical tricuspid repair, the patient underwent successful TEE- and fluoroscopy-guided percutaneous closure of the Gerbode defect using a 6-mm atrial septal defect (ASD) Amplatzer occluder device (Abbott; this device was chosen because the membranous part of the septum was thin). The precise location of the defect was the membranous part of the septum under the noncoronary cusp of the aortic valve.

**Figure 2:**
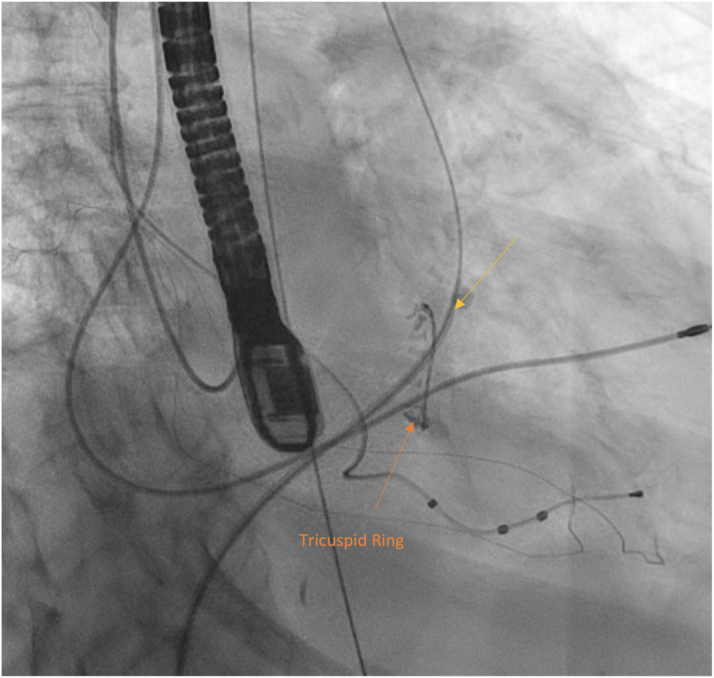
Fluoroscopic Image Demonstrating the Wire and the Sheath (Yellow Arrow) Across the Defect From the Right Ventricular Side (Right Side of the Figure) to the Aorta (Left Side of the Figure)

Given the defect's proximity to the noncoronary cusp, a unique catheter strategy was employed through the femoral artery: A 5-F Judkins right catheter was advanced through a 6-F Judkins right guide catheter to help with directionality. Multiple attempts were made to wire the defect. Ultimately, the defect was wired with a Glidewire (Terumo), which was snared and externalized through the right common femoral vein. An Arrow balloon tip catheter (Teleflex) was then advanced into the LVOT and inflated to see the relation between the defect and the noncoronary cusp, as well as to confirm the septal thickness and defect size (Video 3). Once confirmed, a 6-F 85-cm R2P destination sheath (Terumo) was passed over the Glidewire (Figure 2). Once the sheath was in the left ventricle from the femoral vein, the Glidewire was removed and replaced with a 0.035-inch Safari wire (Lake Region Medical) into the left ventricular apex. This allowed a better coaxial position to deploy the closure device.

After deployment, the device was confirmed to be well seated in stable position with discs straddling the septal defect appropriately (Video 4, Video 5, Video 6, Video 7, Video 8, Video 9). No complications occurred during the procedure, and there was no hemodynamically significant residual shunt (Figure 1 and Video 5). There was no new valvular regurgitation, and the aortic valve and tricuspid valve leaflets were intact and mobile.

Postprocedurally, the patient was prescribed aspirin daily and Eliquis (apixaban) 5 mg twice a day. She was discharged the day of her procedure to start a cardiac rehabilitation program. At follow-up 22 days after the Gerbode defect repair, the patient had significant improvement in her symptoms and no longer required oxygen.

## Discussion

Acquired Gerbode defects, characterized by a left ventricular–to–right atrial shunt through the membranous septum, are uncommon complications of cardiac surgery and other invasive procedures.^1^ The most common mechanism leading to the development of Gerbode defect after tricuspid valve surgical repair is iatrogenic injury to the membranous interventricular septum during suture placement for annuloplasty or valve repair.^1^ The membranous septum lies in close proximity to the tricuspid annulus, particularly near the septal leaflet attachment, making it vulnerable to inadvertent perforation during suture annuloplasty or ring implantation. This creates a direct communication between the left ventricle and right atrium.

The present case illustrates several key challenges and advances in the diagnosis and management of this entity. Notably, the defect remained undetected for over 4 years owing to mischaracterization on TTE as severe tricuspid regurgitation—a diagnostic pitfall well documented in the literature.^1^^,^^7^ This underscores the importance of high clinical suspicion and the use of advanced imaging modalities for accurate diagnosis.

TEE, especially with 3-dimensional (3D) color Doppler and spectral Doppler imaging, played a key role in identifying the location and shape of the defect in this patient, which was situated in the membranous septum beneath the noncoronary cusp of the aortic valve. The value of real-time 3D TEE in accurately showing intracardiac shunts and guiding percutaneous interventions is gaining recognition, as it offers superior anatomical detail and procedural guidance as compared to conventional imaging.^1^^,^^7^

Percutaneous closure of Gerbode defects has emerged as a safe and effective alternative to surgery, especially for patients with suitable anatomy and/or at high surgical risk.^3^, ^4^, ^5^^,^^8^, ^9^, ^10^ In this case, using fluoroscopy and TEE to deploy a 6-mm Amplatzer ASD occluder device allowed successful closure of the defect without complications. The literature supports the use of various occluder devices, such as Amplatzer ASD, VSD, and duct occluders, for Gerbode defects. These devices show high technical success rates, resolution of symptoms, and low complication rates.^3^, ^4^, ^5^^,^^8^, ^9^, ^10^ We selected the Amplatzer ASD occluder because its double-disc design with a self-centering waist is well suited for membranous septal defects beneath the aortic valve. The device apposes the septal wall on each side of the defect, creating a platform for tissue ingrowth while the waist fills the defect diameter. This configuration allows effective sealing of the abnormal left ventricle–to–right atrium communication without impinging on the adjacent aortic, mitral, or tricuspid valves when properly positioned. A multidisciplinary approach with experienced cardiac imagers and interventional cardiologists is key to having a good technical and clinical outcome. Selecting the right device and accompanying catheter strategy for successful device deployment should be tailored to each patient based on the defect's size, location, and proximity to valvular structures.^9^^,^^10^

Although rare, complications such as device embolization, heart block, and valvular dysfunction have been reported after percutaneous closure of Gerbode defects, highlighting the importance of careful planning and monitoring both during and after the procedure.^5^^,^^8^, ^9^, ^10^ In large case series, most patients achieve complete or nearly complete shunt closure, along with significant symptomatic improvement at follow-up.^9^^,^^10^ Our case contributes to the growing evidence supporting percutaneous closure as a primary option for acquired Gerbode defects in properly chosen patients.

## Conclusions

This unique case underscores the diagnostic difficulties and necessary individualized procedural strategies in managing acquired Gerbode defects. Misinterpretation of TTE findings can delay diagnosis, while comprehensive TEE, including 3D and Doppler imaging, is helpful for accurate defect characterization and procedural guidance.^3^^,^^7^ Percutaneous closure of the defect with a septal occluder device proved to be both safe and effective, aligning with current evidence supporting transcatheter approaches for Gerbode defects.^3^, ^4^, ^5^^,^^8^, ^9^, ^10^ Early detection and intervention are important to prevent worsening symptoms and optimize outcomes. Continued reporting of such cases will further inform best practices and device selection for this uncommon but clinically significant complication.

**Visual Summary:**
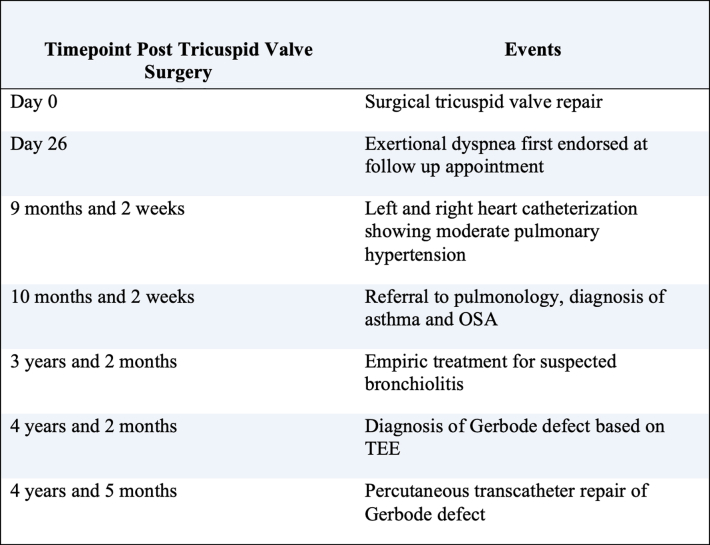
Clinical Event Timeline OSA = Obstructive sleep Apnea; TEE = Transesophageal Echocardiography.

## Funding Support and Author Disclosures

The authors have reported that they have no relationships relevant to the contents of this paper to disclose.

## Appendix

Video 1 Transthoracic Echocardiography 4-Chamber View With Color Doppler Performed 2 Years After Surgical Tricuspid Valve Repair Demonstrating the Defect, Which Was Misread as Tricuspid Valve Regurgitation

Video 2 Transthoracic Echocardiography Zoomed-In 5-Chamber View Performed 3 Years After Surgical Tricuspid Valve Repair Demonstrating the Defect, Which Was Again Misread as Severe Tricuspid Valve Regurgitation

Video 3 Intraprocedural Transesophageal Echocardiography With Color Doppler Illustrating Balloon Sizing of the Defect

Video 4 Fluoroscopy Clip Demonstrating Deployment of the Left Ventricular Side of the Plug

Video 5 Intraprocedural Transesophageal Echocardiography Illustrating the Plug Across the Defect With Elimination of the Flow

Video 6 Intraprocedural Transesophageal Echocardiography Multiplanar Reconstruction View Illustrating the Plug Across the Defect From Different Angles

Video 7 Intraprocedural Transesophageal Echocardiography Illustrating the Plug Being Stable After the Release

Video 8 Fluoroscopy Clip Demonstrating Tag Testing of the Device to Assess Stability

Video 9 Fluoroscopy Clip Demonstrating the Device in Stable Position After Deployment
